# Evaluating medical students’ engagement and confidence across three simulation-based education methods: standardized patient, high fidelity simulator, and virtual reality

**DOI:** 10.1186/s12909-026-08634-9

**Published:** 2026-01-23

**Authors:** Jihye Yu, Sukyung Lee, Mira Kim, Janghoon Lee, Yun Jung Jung

**Affiliations:** 1 Department of medical education, Ajou university school of medicine, Suwon, South Korea; 2 Ajou Center for Clinical Excellence, Ajou University School of Medicine, Suwon, South Korea; 3 Office of medical education, Ajou university school of medicine, Suwon, South Korea; 4 Department of Pediatrics, Ajou university school of medicine, Suwon, South Korea; 5 Department of Pulmonary and Critical Care Medicine, Ajou university school of medicine, Suwon, South Korea

**Keywords:** Simulation-based education, Medical student, Engagement, Clinical confidence

## Abstract

**Background:**

Simulation-based education is a crucial element of medical training, providing safe and realistic environments to develop clinical skills and confidence. This study evaluates the effects of three key simulation methods—Standardized Patients (SP), High-Fidelity Simulators (HFS), and Virtual Reality (VR)—on medical students’ engagement and clinical performance confidence.

**Methods:**

The study involved 43 fifth-year medical students from the Ajou University School of Medicine during a respiratory clinical rotation. The students participated in SP-, HFS-, and VR-based simulation education. After each simulation module, their engagement (interest, flow, relevance) and clinical confidence (history taking, physical examination, interacting and communicating with patients, clinical reasoning) were assessed using self-report surveys. A Multivariate Analysis of Variance was conducted to analyze differences in engagement and confidence across the simulation modules.

**Results:**

The simulation methods had little significant effect on students’ learning engagement or its sub-factors. However, there were significant differences in clinical performance confidence depending on the simulation module. Confidence in history taking, physical examination, and interacting and communicating with patients were significantly higher when using SP compared to HFS and VR.

**Conclusion:**

This study provides practical guidance for designing and developing clinical training curricula by directly comparing the effects of various simulation-based education methods. The results suggest that SP-based simulation education is particularly effective in enhancing medical students’ confidence in clinical settings. While VR and HFS offer advantages in immersion and realism, the experience of interacting with actual patients remain crucial for boosting clinical performance confidence. These findings offer crucial insights for optimizing medical education and improving clinical competencies of medical students.

## Background

Simulation-based education—a crucial component in medical education—aims to enhance the competencies of healthcare professionals, ensure the quality of care, and promote patient safety [1, 2]. Owing to the risks associated with patient safety and privacy, students cannot directly perform clinical procedures on actual patients, which limits their ability to develop clinical skills [3, 4]. Simulation education provides students with environments that closely mimic real clinical settings, enabling them to experience clinical treatments in a realistic manner [5]. Clinical practice conducted via virtual-simulation equipment, with virtual or standardized patients, or in simulated practice rooms, has significantly impacted students’ learning environments and methods [2]. Simulation-based education enables students to experience scenarios that are otherwise rare or pose high risks, thereby improving their knowledge, skills, and attitudes [6]. It provides a realistic learning environment where educators can control the learning environment, offer feedback, and minimize or introduce environmental distractions. Students can experience real-life situations in a safe and supervised setting without risking patient safety [3]. Simulation education provides medical students with various real-life treatment experiences, which enhances their confidence and clinical competence [7, 8] and promotes their learning and professional development [9]. Evidence suggests that providing opportunities for students to practice skills as realistically as possible reduces anxiety during practice, enhances the ability to perform skills, and improves overall learning outcomes [10]. Therefore, it is essential to provide students learning opportunities in an authentic environment where they can test their knowledge and skills without inhibitions [11]. The major simulation methods applied in clinical practice education at medical schools—Standardized Patients (SP), High-Fidelity Simulator (HFS), and Virtual Reality (VR)-based simulation—each have unique educational advantages and provide clinical scenarios to students in various ways. The SP method offers meaningful learning experiences by providing realistic clinical situations through interactions with real patients [12, 13]. High-Fidelity Simulators use highly sophisticated machines and devices that reflect various physiological responses of real patients, such as tactile pulses and auditory breathing sounds [14]. With recent technological advancements, VR simulation has emerged as a promising educational technology owing to its improved cost-effectiveness and user experience [15]. VR realistically represents real-world situations, enabling students’ immersion in standardized clinical situations that are difficult to replicate in actual clinical education [16, 17].

This study aims to analyze the impact of these simulation education methods on medical students’ learning engagement and clinical performance confidence. Engagement is a situation-dependent state involving concentrated participation in a task, encompassing cognitive, behavioral, and emotional aspects. It refers to the will and energy to refocus attention on the task despite distractions [18]. Learning engagement plays a crucial role in the learning experience, directly affecting the quality and outcomes of learning [19, 20]. Engagement is essential for acquiring and understanding knowledge, enabling students to provide effective patient care in clinical situations [21]. Clinical performance confidence impacts students’ ability to confidently perform in real clinical situations, significantly impacting patient safety and care quality [22, 23]. Considering the importance of simulation education in clinical practice education at medical schools to train prospective healthcare professionals for patient safety and quality improvement, it is crucial to compare students’ engagement and confidence across various simulation education methods. This study aims to provide practical guidelines for selecting appropriate educational methods in clinical practice education by analyzing the impact of three simulation-based education methods on learners’ engagement and clinical performance confidence. The specific research questions based on the study’s objectives are as follows:

(1) Is there a difference in self-reported engagement among medical students in SP-, HFS-, and VR-based simulation education?
(2) Is there a difference in self-reported clinical confidence among medical students in SP-, HFS-, and VR-based simulation education?

## Methods

### Design

This study conducted quantitative research through a self-reported survey to examine the impact of simulation-based education experiences, using SP, HFS, and VR, on the engagement and confidence of participating medical students.

### Ethical consideration

This study was approved by the Institutional Review Board (IRB) of Ajou University Hospital (Ethics consent No. AJOUIRB-DB-2024-392).

### Setting

This study was conducted with fifth-year medical students during their internal medicine rotation—specifically, the respiratory medicine segment—from January 30 to April 7, 2023. The study focused on simulation-based education using SP, HFS, and VR. During the one-week respiratory rotation, the education was conducted in the order of HFS, SP, and VR.

The HFS used Laerdal’s SimMan simulator and was conducted in groups of 2–3 students. The simulation scenario focused on acute respiratory distress. After an orientation on the simulation environment, procedure, and duration, students engaged in the 15-minute simulation based on the given scenario. The learning objectives of the HFS session were to (1) identify signs of acute respiratory distress, (2) perform appropriate initial interventions, (3) make rapid clinical decisions, and (4) manage the scenario collaboratively within a small team.

In the SP-based simulation, students conducted one-on-one patient interviews and performed clinical assessments with standardized patients. The case involved a tuberculosis patient presenting with a chief complaint of coughing. After a 12-minute patient interview, students documented 3–4 likely diagnoses, required diagnostic plans (tests and physical examinations), and treatment plans based on the interview. The SP-based simulation aimed to develop students’ competencies in (1) conducting focused history-taking, (2) performing relevant physical examinations, (3) formulating differential diagnoses, and (4) practicing patient-centered communication.

The VR-based simulation utilized Sim-X and Oculus Quest 2. Students used handheld controllers to detect movements and interact with virtual objects. The simulation was conducted in groups of 2–3 students and was based on a sepsis scenario. Following a preliminary explanation and practice session on using the VR equipment, students participated in a 15-minute VR simulation. After each simulation session, a 30-minute group debriefing, including feedback from the instructor, was conducted. The VR simulation focused on (1) recognizing early signs of sepsis, (2) prioritizing clinical decision-making in a time-sensitive situation, (3) interacting with virtual patients and equipment, and (4) applying step-by-step clinical reasoning within a virtual environment.

### Participants

This study analyzed self-reported survey results from 43 fifth-year medical students (31 males and 12 females) at the Ajou University School of Medicine in 2023. The analysis included all responses without any missing data.

### Data collection tools and study procedure

In this study, the impact of each simulation-based educational experience on learners was assessed by measuring their engagement and confidence. Engagement was measured using self-reported surveys distributed after each simulation module. To evaluate medical students’ engagement, we utilized eight items from the Exit Survey developed by McCoy [24] and specifically used by McCoy [25] for measuring engagement. McCoy’s engagement sub-scale includes two items for relevance, two items for interest, and four items for flow. Relevance items measured the connection to real clinical situations, applicability, and relevant feedback. Interest items measured enthusiasm and passion, while flow items measured immersion and concentration. Each item was rated on a 5-point Likert scale ranging from 1 (strongly disagree) to 5 (strongly agree). The evaluation scores were calculated as the average of the item scores within each domain. In this study, the Cronbach’s α for student engagement after experiencing the three simulation education modules was 0.866 for SP-based, 0.863 for HFS-based, and 0.918 for VR-based simulations.

Confidence was measured using four items adapted from Ytterberg et al. [26] that were relevant to this study’s context. These survey items measured confidence levels in history-taking, physical examination, interacting and communicating with patients, and clinical reasoning, using a 10-point rating scale. The survey was administered as a self-reported questionnaire both before and after the three simulation practice sessions. The pre-simulation Cronbach’s α for confidence was 0.813. After experiencing the simulation education modules, it was 0.910 for SP-based, 0.866 for HFS-based, and 0.935 for VR-based simulations.

### Data analysis

Statistical analysis was performed using SPSS Statistics 24. To analyze the differences in engagement, confidence, and their sub-factors according to the simulation education methods, a Multivariate Analysis of Variance (MANOVA) was conducted. Although engagement and confidence scores were derived from Likert-type survey items, the aggregated scale scores were treated as approximately continuous because composite Likert scales are commonly analyzed using parametric methods in educational and psychometric research. Considering this established analytic practice and the multivariate nature of the variables, MANOVA was deemed an appropriate statistical approach for this study.

## Results

The results of investigating the impact of various simulation methods (SP, HFS, VR) on engagement and its sub-factors (relevance, interest, flow) are presented in Table 2  and  Table 1. The MANOVA revealed that the multivariate effect of simulation methods on the dependent variables was significant (Wilks’ Lambda = 0.842, F(8, 246) = 2.762, *p* < .01). This indicates statistically significant differences in engagement and its sub-factors depending on the different simulation methods.

**Table 1:** Multivariate analysis of variance results for engagement

Effect	Wilks’ Lambda	F	df1	df2	*p*-value
Simulation method	0.842	2.762	8	246	< 0.01

**Table 2:** Univariate ANOVA results for each dependent variable related to engagement

Dependent Variable	Group	M ± SD	F	df	*p*-value
Engagement	SP	4.54 ± 0.56	0.482	2	0.618
	HFS	4.42 ± 0.56			
	VR	4.30 ± 0.70			
Relevance	SP	4.45 ± 0.48	1.379	2	0.256
	HFS	4.54 ± 0.40			
	VR	4.45 ± 0.53			
Interest	SP	4.28 ± 0.61	2.167	2	0.119
	HFS	4.48 ± 0.44			
	VR	4.36 ± 0.60			
Flow	SP	4.52 ± 0.53	1.647	2	0.197
	HFS	4.71 ± 0.40			
	VR	4.70 ± 0.45			

The analysis results for engagement showed no significant effect of the simulation methods (F = 0.48, p = .618). The mean engagement scores were 4.54 (SD = 0.56) for SP, 4.42 (SD = 0.56) for HFS, and 4.30 (SD = 0.70) for VR, indicating similar levels of engagement across the three simulation methods. The analysis of variance for relevance also revealed no significant effect of the simulation methods (F = 1.38, p = .256). The mean relevance scores were 4.45 (SD = 0.48) for SP, 4.54 (SD = 0.40) for HFS, and 4.45 (SD = 0.53) for VR, suggesting no significant differences among the different methods. Similarly, the analysis for flow showed no significant effect of the simulation methods (F = 1.65, p = .197). The mean flow scores were 4.52 (SD = 0.53) for SP, 4.71 (SD = 0.40) for HFS, and 4.70 (SD = 0.45) for VR.

In summary, the MANOVA results suggested a significant impact of the simulation methods on engagement and its sub-factors. However, subsequent one-way ANOVA analyses revealed no significant differences in individual engagement or its sub-factors—relevance, interest, and flow. This indicates that while simulation methods influence engagement in a multivariate context, no significant differences were identified when analyzing each element separately.

The results of analyzing the impact of SP, HFS, and VR on medical students’ clinical confidence are presented in  Table 3. The investigation into how the simulation methods (SP, HFS, VR) affect the four areas of clinical confidence (history-taking, physical examination, interacting and communicating with patients, and clinical reasoning) showed statistically significant differences in clinical confidence and its sub-factors depending on the different simulation methods (Wilks’ Lambda = 0.836, F(8, 246) = 2.885, *p* < .01).

**Table 3:** Multivariate analysis of variance results for clinical confidence

Effect	Wilks’ Lambda	F	df1	df2	*p*-value
Simulation method	0.836	2.885	8	246	< 0.01

The results of analyzing the impact of simulation methods on each dependent variable are presented in Table 4. There were significant differences based on the simulation method for history-taking (F = 8.569, p < .001), physical examination (F = 6.110, p = .003), and interacting and communicating with patients (F = 6.866, p = .001). However, there were no significant differences for clinical reasoning (F = 1.570, p = .212). 

**Table 4:** Univariate ANOVA results for each dependent variable related to clinical confidence

Dependent Variable	Group	M ±SD	F	df	p-value
History-taking	SP	7.19±1.18	8.569	2	.000
	HFS	6.21±1.36			
	VR	5.95±1.77			
Physical examination	SP	6.79±1.28	6.110	2	.003
	HFS	5.93±1.32			
	VR	5.81±1.62			
Interacting and communicating with patients	SP	7.60±1.24	6.866	2	.001
	HFS	6.51±1.50			
	VR	6.53±1.88			
Clinical reasoning	SP	6.44±1.24	1.570	2	.212
	HFS	5.93±1.28			
	VR	6.02±1.71			

The post-hoc analysis results for differences in clinical confidence based on the simulation methods are presented in Table 5.

**Table 5:** Post-hoc pairwise comparisons for clinical confidence using Tukey HSD

Dependent Variable	Comparison (I vs. J)	Mean Difference (I-J)	Std. Error	*p*-value
History-taking	SP vs. HFS	0.98	0.314	0.007
	SP vs. VR	1.23		0.000
	HFS vs. VR	0.26		0.695
Physical examination	SP vs. HFS	0.86	0.305	0.015
	SP vs. VR	0.98		0.005
	HFS vs. VR	0.12		0.923
Interacting and communicating with patients	SP vs. HFS	1.09	0.337	0.004
	SP vs. VR	1.07		0.005
	HFS vs. VR	− 0.02		0.997
Clinical reasoning	SP vs. HFS	0.51	0.308	0.223
	SP vs. VR	0.42		0.365
	HFS vs. VR	− 0.09		0.951

The SP simulation group scored significantly higher than the HFS and VR simulation groups in the areas of history-taking, physical examination, and interacting and communicating with patients. Conversely, there were no significant differences between the HFS and VR simulation groups in these areas. Additionally, no significant differences were identified among the three groups in clinical reasoning. These results suggest that clinical confidence can vary in specific areas depending on the simulation method applied. Particularly, SP-based simulation training appears to be more effective for history-taking, physical examination, and interacting and communicating with patients.

## Discussion

This study aimed to compare and analyze the effects of simulation-based clinical practice education using SP, HFS, and VR on medical students’ engagement and clinical confidence. The results indicated that students demonstrated high levels of engagement across all three simulation modules, with no significant differences in engagement levels based on the simulation method. This suggests that each method effectively maintains student engagement. Although the MANOVA indicated statistically significant multivariate differences in engagement across simulation types, the subsequent univariate analyses did not reveal significant differences in individual engagement components (relevance, interest, flow). This suggests that the combined multivariate pattern of engagement differed subtly across modalities, but the effect was not strong enough for any single engagement variable to reach statistical significance when analyzed independently. These findings imply that intercorrelations among engagement components contributed to the multivariate significance detected by MANOVA.

Although previous studies [17, 18, 25, 27] have suggested that VR enhances concentration, immersion, and engagement more than other simulation methods, our study identified no significant differences in engagement when comparing VR to SP and HFS simulations. A study comparing hybrid (actor, manikin) and VR simulations [19] reported that VR did not increase student engagement, with traditional simulation environments using actors and manikins showing higher engagement. This discrepancy may be due to the complexity of operating VR simulations, despite prior training on VR equipment.

These findings imply that brief pre-training sessions may not fully address usability issues, highlighting the need for more extensive preparation to acclimate students to the virtual environment. While VR holds promising technical advantages, it currently has limitations in fully replicating real clinical scenarios. Issues such as a sense of disconnection from reality, discomfort from VR headsets, and dizziness could hinder student participation and learning [17, 28]. These results suggest that despite technological advancements, VR cannot completely replace the dynamic and natural experiences obtained through real patient interactions.

Additionally, our study confirmed that HFS- and SP-based simulations are effective methods for fostering student interest, immersion, and engagement. The high realism provided by HFS-based simulations and the authentic patient interactions offered by SP-based simulations encourage active student participation. Medical students value realistic interactions with live patients [29], and recognizing the importance of natural human interaction in traditional simulation methods is essential for fostering student engagement [19].

There were significant differences in students’ clinical confidence based on the simulation method. SP-based simulations were more effective than HFS- and VR-based simulations in enhancing confidence in history-taking, physical examination, and interacting and communicating with patients. It is also important to note that the SP session specifically targeted skills such as history-taking, physical examination, and patient communication, whereas the HFS and VR sessions focused on different clinical objectives. Therefore, part of the difference in confidence scores may be related to differences in learning objectives across the three simulations. This suggests that the real patient interaction experience provided by SP can improve students’ clinical confidence. Conversely, there were no significant differences in confidence between the VR and HFS methods.

Previous studies on clinical confidence in high- and low-fidelity simulators have shown mixed results. A few studies reported no significant differences in confidence between high- and low-fidelity simulators [30, 31, 32]. Others suggested that high-fidelity simulators positively impact self-confidence, self-efficacy, critical thinking, and clinical judgment [33]. These conflicting findings indicate that different approaches may be necessary depending on educational goals and learner needs.

HFS are advantageous for practicing detailed technical skills and managing complex clinical scenarios. Previous studies have also suggested that SP-based simulations can support the assessment of clinical performance [34, 35] and may help improve communication skills in a safe environment. They may further provide meaningful learning experiences through interaction and feedback with standardized patients [12]. Therefore, educators should consider the unique educational strengths of each simulation modality and select appropriate methods based on students’ learning objectives and instructional contexts.

This study contributes to understanding the impact of various simulation-based clinical practice education methods on medical students’ engagement and clinical confidence. While many studies investigate the effectiveness of individual simulation modalities, comparative studies of different simulation methods are relatively rare. Such comparative research provides novel insights and opportunities to comprehensively analyze the strengths and weaknesses of various educational approaches.

Furthermore, the findings offer practical guidance for designing and developing medical school curricula. If SP-based simulations have been described as more effective in history-taking, physical examination, and patient interaction, optimizing their use and integrating them with other modalities is advisable. Similarly, VR and HFS are more effective for understanding technical details and complex clinical scenarios [14, 25, 33], underscoring the importance of utilizing these methods to develop comprehensive educational programs.

This study has a few limitations. First, the sample was limited to medical students from a single university, potentially restricting the generalizability of the findings. Second, the data were collected via self-reported surveys, which may rely on subjective evaluations and differ from objective assessment results. Third, all students completed the three simulation modalities in the same fixed sequence (HFS → SP → VR), raising the possibility of order effects. Participation in earlier sessions may have influenced students’ engagement or confidence in the later sessions. Future studies should consider counterbalancing or randomizing the order of the simulation modalities using all possible combinations. Fourth, the study period was brief, limiting the evaluation of long-term educational effects. Future research should include larger samples from multiple universities, use various evaluators and objective assessment tools, and assess long-term educational impacts. Fifth, MANOVA was applied to data originating from Likert-type ordinal scales. Although composite scores are commonly analyzed as continuous variables in educational research, this approach may not fully reflect the ordinal nature of the data. Future research could consider non-parametric or alternative statistical methods to validate these findings.

In conclusion, this study compared and analyzed the effects of SP-, HFS-, and VR-based simulation education on medical students’ engagement and clinical confidence. The findings showed that all three simulation methods effectively maintained student engagement. SP-based simulations provided higher clinical performance confidence in history-taking, physical examination, and patient interaction and communication, while no significant differences in confidence were observed between VR and HFS.

These results suggest the need to appropriately integrate various simulation methods in medical school clinical practice curricula. Leveraging the strengths of SP-based simulations to enhance clinical performance confidence, while incorporating the technical advantages of HFS and VR, could provide high-quality clinical practice education. Such an approach will help medical students develop the competencies required to effectively manage diverse clinical situations.

## Conclusion

This study examined the impact of SP-, HFS-, and VR-based simulation education on medical students’ engagement and clinical confidence. The results demonstrate that all three simulation methods effectively maintain student engagement, while SP-based simulations significantly enhance clinical confidence in history-taking, physical examination, and patient interaction. These findings highlight the need to strategically integrate diverse simulation methods into clinical practice curricula, leveraging the realistic patient interactions of SP-based simulations alongside the technical advantages of HFS and VR. By combining these approaches, medical educators can provide comprehensive and high-quality training, equipping students with the skills and confidence necessary to excel in diverse clinical scenarios and improve patient care outcomes. Future research should focus on expanding participant diversity, incorporating objective assessments, and evaluating long-term impacts to further refine simulation-based education.

## Data Availability

The datasets are available from the corresponding author on reasonable request.
